# Structural comparison of CD163 SRCR5 from different species sheds some light on its involvement in porcine reproductive and respiratory syndrome virus-2 infection in vitro

**DOI:** 10.1186/s13567-021-00969-z

**Published:** 2021-06-30

**Authors:** Hongfang Ma, Rui Li, Longguang Jiang, Songlin Qiao, Xin-xin Chen, Aiping Wang, Gaiping Zhang

**Affiliations:** 1 School of Life Sciences, Zhengzhou University, Zhengzhou, 450001 Henan China; 2 Key Laboratory of Animal Immunology of the Ministry of Agriculture, Henan Provincial Key Laboratory of Animal Immunology, Henan Academy of Agricultural Sciences, Zhengzhou, 450002 Henan China; 3 College of Chemistry, Fuzhou University, Fuzhou, 350116 Fujian China; 4 College of Veterinary Medicine, Henan Agricultural University, Zhengzhou, 450002 Henan China; 5 Jiangsu Co-Innovation Center for the Prevention and Control of Important Animal Infectious Diseases and Zoonoses, Yangzhou University, Yangzhou, 225009 Jiangsu China

**Keywords:** PRRSV, CD163, SRCR5, Crystal structure, Infection

## Abstract

Porcine reproductive and respiratory syndrome (PRRS) is a serious disease burdening global swine industry. Infection by its etiological agent, PRRS virus (PRRSV), shows a highly restricted tropism of host cells and has been demonstrated to be mediated by an essential scavenger receptor (SR) CD163. CD163 fifth SR cysteine-rich domain (SRCR5) is further proven to play a crucial role during viral infection. Despite intense research, the involvement of CD163 SRCR5 in PRRSV infection remains to be elucidated. In the current study, we prepared recombinant monkey CD163 (moCD163) SRCR5 and human CD163-like homolog (hCD163L1) SRCR8, and determined their crystal structures. After comparison with the previously reported crystal structure of porcine CD163 (pCD163) SRCR5, these structures showed almost identical structural folds but significantly different surface electrostatic potentials. Based on these differences, we carried out mutational research to identify that the charged residue at position 534 in association with the one at position 561 were important for PRRSV-2 infection in vitro. Altogether the current work sheds some light on CD163-mediated PRRSV-2 infection and deepens our understanding of the viral pathogenesis, which will provide clues for prevention and control of PRRS.

## Introduction

PRRS is characterized by reproductive failures in sows of late-term gestation and respiratory signs in pigs of all ages. It causes significant economic losses to global swine industry [1, 2]. PRRS has an estimated annual cost of $664 million in the USA alone [3]. PRRSV, as the causative agent, is an enveloped single-stranded positive-sense RNA virus [4, 5] and belongs to the genus *Betaarterivirus*, family *Arteriviridae*, order *Nidovirales* [6, 7]. All PRRSV isolates are classified into PRRSV-1 and PRRSV-2 [8, 9]. In China, PRRSV-2 strains are predominantly prevalent [10, 11], and highly pathogenic variants of PRRSV-2 (HP-PRRSVs) result in serious declines in pig production throughout the country [12, 13].

PRRSV infection shows a strongly restricted tropism for host species and target cells. Swine, including domestic pigs and wild boar, are the only known hosts for PRRSV [14–19]. CD163-positive macrophages, particularly porcine alveolar macrophages (PAMs), are PRRSV primary target cells in vivo [20]. In addition, African green monkey kidney epithelial cell MA-104 and its derivatives, MARC-145 and CL2621 cells, support viral infection in vitro [16, 21]. The specific tropism of PRRSV is mediated by host cell receptor(s) [22–24]. Stable expression of SR CD163 from different species (pig, human and monkey) was found to render various non-permissive cells susceptible to PRRSV infection, including porcine kidney (PK 032495), Norden Laboratories feline kidney (NLFK) and baby hamster kidney (BHK-21) cells [25]. Other cell lines, such as SV40-transformed PAM 3D4/21 (CRL-2843) [26], PK-15 [27, 28] and murine macrophage-derived cell lines [29], were also susceptible to PRRSV with expression of pCD163. Recent studies have demonstrated that gene-edited pigs lacking functional pCD163 are completely resistant to PRRSV, which confirms that it is an indispensable receptor for the viral infection [30–33].

CD163 contains nine class B SRCR domains (SRCR1-9) in its large ectodomain [34, 35]. SRCR5 has been shown to play a crucial role during PRRSV infection in vitro [36–38]. Additionally, a significantly reduced permissiveness to PRRSV-2, particularly HP-PRRSV, was shown in the PAMs with pCD163 SRCR5 substitution by homologous hCD163L1 SRCR8 [31, 39]. Despite these studies, the mechanisms by which CD163 SRCR5 takes effect in PRRSV infection are not fully elucidated.

In this work, we prepared recombinant moCD163 SRCR5 in *Pichia pastoris* X-33 and hCD163L1 SRCR8 in *Drosophila melanogaster* Schneider 2 (S2) cells, respectively. After purification and crystallization, we determined their crystal structures and aligned them with that of pCD163 SRCR5 we previously reported [40]. Based on the structural comparison, we carried out mutational assays to explore which residues are important for PRRSV-2 infection and how they influence the viral infection in vitro.

## Materials and methods

### Materials, cell lines and viruses

All chemicals were purchased from Sigma-Aldrich Co., Ltd. (St. Louis, USA) or Sinopharm Chemical Reagent Co., Ltd. (Shanghai, China) unless otherwise stated.

The *Drosophila* S2 cells were kept in Schneider’s insect medium supplemented with 10% heat-inactivated fetal bovine serum (FBS; Gibco, Grand Island, USA) and antibiotics (100 U/mL penicillin, 100 μg/mL streptomycin; Gibco) at 28 °C in a humidified incubator. The PK-15 cells and MARC-145 cells were maintained routinely in Gibco Dulbecco’s modified Eagle’s medium (DMEM) supplemented with 10% heat-inactivated FBS and antibiotics at 37 °C in a humidified incubator with a 5% CO_2_ atmosphere.

The PRRSV-2 strain BJ-4 (Genbank ID: AF331831) was isolated on MARC-145 cells in China and kindly provided by Professor Hanchun Yang of China Agricultural University [41]. The HP-PRRSV strain HN07-1 (Genbank ID: KX766378.1) was isolated on PAMs in Henan Province of China by our laboratory [42].

### Preparation of moCD163 SRCR5 in *Pichia pastoris* X-33 cells

MoCD163 SRCR5 was prepared in *Pichia pastoris* X-33 cells according to our previous studies [43, 44]. Briefly, the cDNA encoding moCD163 SRCR5 (residues 478-578, the numbering is according to UniProt entry Q2VLG4) was synthesized by Shanghai Sangon Biotech Co., Ltd. (Shanghai, China) and inserted into the expression vector pPICZαA (Invitrogen, Carlsbad, USA) between the *Xho* I and *Sal* I sites. The recombinant moCD163 SRCR5 expression vector was verified by Shanghai Sangon Biotech Co., Ltd., linearized using *Pme* I (New England Biolabs, Ipswich, USA) and transformed into competent X-33 cells by electroporation (1500 V, 25 μF, 200 Ω for 6 ms). For a large scale expression, recombinant X-33 cells were first grown in buffered minimal glycerol-complex medium (BMGY) (1% yeast extract, 2% peptone, 100 mM potassium phosphate pH 6.0, 1.34% yeast nitrogen base (YNB), 4 × 10^–5^% biotin, 1% v/v glycerol) to an OD_600_ reading of 2–6 and then in buffered minimal methanol-complex medium (BMMY) (1% yeast extract, 2% peptone, 100 mM potassium phosphate pH 6.0, 1.34% YNB, 4 × 10^–5^% biotin, 1% v/v methanol). The protein expression was induced every 24 h with 1% (v/v) methanol for 4 days.

### Preparation of hCD163L1 SRCR8 in *Drosophila* S2 cells

The cDNA encoding hCD163L1 SRCR8 (residues 795-895, the numbering is according to UniProt entry Q9NR16) was synthesized by Shanghai Sangon Biotech Co., Ltd. and inserted into the expression vector pMT/BiP/V5-HisA (Invitrogen) between the *Bgl* II and *Mlu* I sites. The recombinant expression vector was verified by Shanghai Sangon Biotech Co., Ltd. and transfected into *Drosophila* S2 cells with the pCoBlast vector by Cellfectin II reagent according to Invitrogen’s instructions. The selected stably transfected *Drosophila* S2 cells were grown in Sf-900 II serum-free medium (Invitrogen) and induced by 0.75 mM CuSO_4_ for five days according to our methods [45, 46].

### Purification of recombinant target proteins

After centrifugation and clarification by filtration, the supernatant containing each target protein was applied to GE Ni Sepharose excel column (Boston, USA) pre-equilibrated with 20 mM Tris–HCl pH 8.0, 150 mM NaCl. Each target protein was then eluted with 20 mM Tris–HCl pH 8.0, 150 mM NaCl and 200 mM imidazole. The eluent containing each target protein was further purified by GE Superdex 200 10/300 GL prepacked column on the GE AKTA Pure system (Uppsala, Sweden) with 20 mM Tris–HCl pH 8.0, 150 mM NaCl as elution buffer. The fraction containing moCD163 SRCR5 or hCD163L1 SRCR8 was collected, dialyzed, and concentrated to 7.75 and 10 mg/mL in 20 mM Tris–HCl pH 8.0, 20 mM NaCl, respectively.

### Crystallization, data collection, and structural determination of target proteins

Crystallization of each target protein was carried out at room temperature (RT; 25 °C) by the sitting-drop vapor diffusion method with an equal volume of each target protein and various crystallization reagents from the Hampton crystallization screening kits (Aliso Viejo, USA). Single crystals of moCD163 SRCR5 were acquired under 0.1 M Bis–Tris pH 5.5, 25% PEG 3350, 0.2 M NaCl, and those of hCD163L1 SRCR8 were acquired under 0.1 M citric acid pH 3.5, 15% PEG 3350. The crystals were flash-frozen in liquid nitrogen using a cryoprotection solution with 20% glycerol in the crystallization solution. X-ray data sets of the crystals were collected at a wavelength of 0.979 Å on the beamlines BL18U and BL19U1 at the Shanghai Synchrotron Radiation Facility (SSRF) [47]. Diffraction data sets were processed using the HKL-3000 package [48]. The crystal structures were solved by molecular replacement using pCD163 SRCR5 (PDB code 5JFB) as the search model [40] with the program Molrep in CCP4 suites [49]. The structures were refined by CCP4 program package and manually adjusted by the molecular graphics program COOT [50]. Solvent molecules were added using a F_o_-F_c_ Fourier difference map at 2.5 σ in the final refinement step. Statistics of data collection and final model refinement were summarized in Table 1. The final structures were analyzed by the software PyMOL [51].

**Table 1:** **X-ray data collection and model refinement statistics**

Crystal	moCD163 SRCR5	hCD163L1 SRCR8
PDB code	6K0L	6K0O
X-ray source wavelength (Å)	0.979	0.979
Resolution limits (Å)	1.58	2.0
Space group	P1	C2
Temperature of experiments (K)	100	100
Cell parameters (Å)	a = 28.6, b = 33.5, c = 46.7	a = 29.5, b = 73.0, c = 82.7
	α = 71.0°, β = 75.3°, γ = 84.9°	α = 90.0°, β = 90.0°, γ = 90.0°
Completeness (%)	95.1 (94.4)^a^	98.9 (96.7)^a^
Redundancy	3.4 (3.6)^a^	5.3 (3.5)^a^
Rmerge^b^	0.040 (0.096)^a^	0.152 (0.378)^a^
I/σ (I)	24.6 (13.6)^a^	13.4 (4.0)^a^
Number of unique reflections	20 604	11 711
CC_1/2_	0.992	0.985
**Refinement data**		
R factor	0.158	0.195
R free	0.193	0.269
r.m.s deviation of bond lengths (Å^2^)	0.007	0.012
r.m.s deviation of angle (°)	1.17	1.64
Ramachandran analysis (%)	98.1^c^, 3.4^d^, 0.5^e^	95.6^c^, 2.9^d^, 1.5^e^

^a^Numbers in parentheses refer to the highest resolution shells.

^b^Rmerge = Σ|*Ii*- < *I* >|/Σ*Ii*, where *Ii* is the intensity of the h observation and < *I* > is the mean intensity of the reflections.

^c^Percentage of residues in most favored regions.

^d^Percentage of residues in additional allowed regions.

^e^Percentage of residues in generously allowed regions.

### Site-directed mutagenesis of SRCR5 in pCD163

We utilized a construct with complete wild-type (WT) pCD163 cDNA integrated into the PiggyBac transposon system (kindly provided by Professor Enmin Zhou, Northwest Agriculture and Forestry University, China) as a template to generate each single-site mutant encoding pCD163 G499R, E509H, S512N, T522D, E534K, E543K, H549S, P560Q, R561H and G564D (the numbering is according to UniProt entry Q2VL90). The primers designed for mutation were listed in Table 2. All mutation constructs were verified by Shanghai Sangon Biotech Co. Ltd.

**Table 2:** **Primers for mutagenesis and RT-qPCR in this study**

Primer	Sequence^a^
Mutagenesis	
pCD163-G499R, forward	GTACAACATGGAGACACGTGGcgcACCGTCTGTGATTCTGAC
pCD163-G499R, reverse	GTCAGAATCACAGACGGTgcgCCACGTGTCTCCATGTTGTAC
pCD163-E509H, forward	GTGATTCTGACTTCTCTCTGcacGCGGCCAGCGTGCTGTGC
pCD163-E509H, reverse	GCACAGCACGCTGGCCGCgtgCAGAGAGAAGTCAGAATCAC
pCD163-S512N, forward	GACTTCTCTCTGGAGGCGGCCaacGTGCTGTGCAGGGAACTAC
pCD163-S512N, reverse	GTAGTTCCCTGCACAGCACgttGGCCGCCTCCAGAGAGAAGTC
pCD163-T522D, forward	GAACTACAGTGCGGCgacGTGGTTTCCCTC
pCD163-T522D, reverse	CAGGAGGGAAACCACgtcGCCGCACTGTAGTTC
pCD163-E534K, forward	CTGGGGGGAGCTCACTTTGGAaaaGGAAGTGGACAGATCTG
pCD163-E534K, reverse	CAGATCTGTCCACTTCCtttTCCAAAGTGAGCTCCCCCCAG
pCD163-E543K, forward	GTGGACAGATCTGGGCTGAAaaaTTCCAGTGTGAGGGGCACGAG
pCD163-E543K, reverse	CTCGTGCCCCTCACACTGGAAtttTTCAGCCCAGATCTGTCCAC
pCD163-H549S, forward	GAAGAATTCCAGTGTGAGGGGagcGAGTCCCACCTTTCACTCTG
pCD163-H549S, reverse	CAGAGTGAAAGGTGGGACTCgctCCCCTCACACTGGAATTCTTC
pCD163-P560Q, forward	CTTTCACTCTGCCCAGTAGCAcaaCGCCCTGACGGGACATGTAGC
pCD163-P560Q, reverse	GCTACATGTCCCGTCAGGGCGttgTGCTACTGGGCAGAGTGAAAG
pCD163-R561H, forward	CACTCTGCCCAGTAGCACCCcacCCTGACGGGACATGTAGCCAC
pCD163-R561H, reverse	GTGGCTACATGTCCCGTCAGGgtgGGGTGCTACTGGGCAGAGTG
pCD163-G564D, forward	CAGTAGCACCCCGCCCTGACgacACATGTAGCCACAGCAGGGAC
pCD163-G564D, reverse	GTCCCTGCTGTGGCTACATGTgtcGTCAGGGCGGGGTGCTACTG
pCD163-E534K/R561H, forward	CGGCGTAGTCTGCTCAAGATACACACAAATC
pCD163-E534K/R561H, reverse	CTCCAACCAGCCTGGGTTTCCTGTGGGCTG
RT-qPCR	
PRRSV-ORF7, forward	AAACCAGTCCAGAGGCAAGG
PRRSV-ORF7, reverse	GCAAACTAAACTCCACAGTGTAA
GAPDH, forward	CCTTCCGTGTCCCTACTGCCAAC
GAPDH, reverse	GACGCCTGCTTCACCACCTTCT

^a^The lowercase letters indicate mutated sites.

### Cell transfection with WT or mutant pCD163

PK-15 cells were seeded at a density of 4.0 × 10^5^ cells/mL and incubated overnight. The PK-15 cells were transfected with the same amount (1 μg/well, 24-well plate or 3 μg/well, 6-well plate) construct of WT or mutant pCD163 using Lipofectamine LTX reagent with Plus reagent according to the manufacturer’s instructions (Invitrogen). The expression levels of WT and each mutant pCD163 were measured by immunofluorescence assay (IFA) or immunoblotting (IB).

### Immunofluorescence assay (IFA)

Cells were grown in 24-well plates, fixed with 4% paraformaldehyde (PFA) for 15 min and permeabilized with 0.1% Triton X-100 in PBS at RT for 5 min. Anti-CD163 antibody (MCA2311GA; AbD Serotec, Hercules, USA), anti-PRRSV nucleocapsid (N) protein antibody (kept in our laboratory) and DAPI were used to stain CD163, PRRSV N protein and nuclei, respectively. Then the cells were stained with the appropriate secondary antibodies. Images were representative as a single slice of a stack from three independent experiments. Quantitative analyses of single channel fluorescence were performed using ImageJ software [52, 53].

### Immunoblotting (IB)

The cells were harvested and lysed in radio immunoprecipitation assay (RIPA) lysis buffer (Beyotime Biotechnology, Shanghai, China). The lysates were normalized to equal amounts of tubulin, separated by sodium dodecyl sulfate polyacrylamide gel electrophoresis, and electro-transferred onto Immobilon-P membranes (Merck Millipore, Darmstadt, Germany). The membranes were blocked in 5% skimmed milk for 1 h, and probed with the mouse anti-human CD163 (MCA1853, AbD serotec), mouse anti-PRRSV N protein or mouse anti-β-tubulin monoclonal antibody (3G6, Abbkine, Wuhan, China). After incubation with horseradish peroxidase (HRP)-labeled goat anti-mouse IgG antibody as secondary antibody, the indicated proteins were visualized by enhanced chemiluminescence (ECL) reagent (Solarbio, Beijing, China).

### Quantitative real-time PCR (RT-qPCR)

Total RNAs from PRRSV-inoculated PK-15 cells were extracted with TRIzol reagent (Invitrogen). The reversely transcribed cDNAs were prepared using the PrimeScript RT reagent kit with gDNA Eraser (TaKaRa, Dalian, China) and amplified by RT-qPCR to measure RNA abundance on a 7500 Fast RT-PCR system (Applied Biosystems, Foster City, USA). PRRSV open reading frame (ORF) 7 gene was normalized with housekeeping glyceraldehyde-3-phosphate dehydrogenase (GAPDH) mRNA and relatively quantified by the 2^−ΔΔCT^ method [54], or quantitated using a plasmid containing PRRSV ORF7 as the template to generate a standard curve to calculate the actual RNA copies [40]. Three replicates were run, and each experiment was independently repeated for three times.

### PRRSV titration assay

The transfected cells were inoculated with PRRSV at a multiplicity of infection (MOI) of 1 and incubated at 37 °C for 3 h. The viruses not entering into the cells were then washed away. At 48 h post-infection (hpi), the progeny virus titers were measured by the 50% tissue culture infected dose (TCID_50_) assay in MARC-145 cells according to Reed and Muench [55].

### PRRSV binding, entry and infection assays

For PRRSV binding assay [56–58], PRRSV strain BJ-4 or HN07-1 at a MOI of 1 was inoculated in the transfected cells at 4 °C for 1 h. After the unbound viruses was washed away, the level of cell-bound viral RNA (PRRSV ORF7) was measured by RT-qPCR. For PRRSV entry assay [59, 60], the unbound viruses were washed away and the inoculated cells were cultured at 37 °C for 3 h to allow viral entry. The viruses not entering into the cells were washed, and the entering viral RNA was analyzed by RT-qPCR. For PRRSV infection assay, the infected cells were further cultured for 9 h, and at 12 hpi, viral RNA abundance was analyzed by RT-qPCR. The PK-15 cells transfected with empty vector were inoculated with PRRSV and assayed in parallel as negative control. Three replicates were run, and each experiment was independently repeated for three times.

### Statistical analysis

All experimental data were presented as group means and standard errors of the means (SEM). The experimental data were analyzed using the unpaired, 2-tailed Student *t* test with Origin software. Differences at the 95% confidence level (*p* < 0.05) were considered statistically significant.

## Results

### Crystal structures of moCD163 SRCR5 and hCD163L1 SRCR8

The recombinant moCD163 SRCR5 and hCD163L1 SRCR8 were eluted as monomer during purification. Their crystal structures were determined and refined to 1.58 Å in P1 space group and 2.0 Å in C2 space group, respectively (Table 1). Both these two target proteins were crystallized with two molecules in each asymmetric unit (data not shown). We analyzed only one representative molecule for each protein since the root mean square deviation (RMSD) differences of the two molecules were slight (0.603 Å for 86 matching Cα atoms of moCD163 SRCR5, 0.068 for 92 matching Cα atoms of hCD163L1 SRCR8).

**Figure 1:**
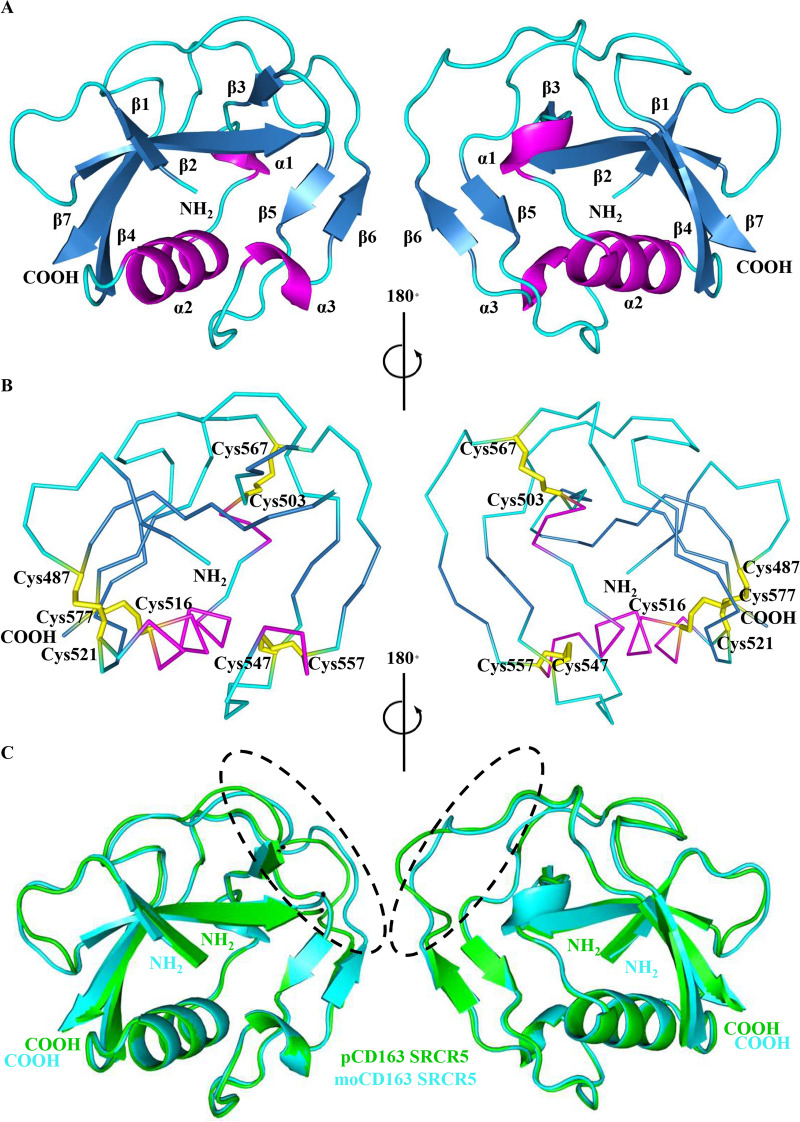
**Crystal structure of moCD163 SRCR5 and comparison with pCD163 SRCR5. A** The cartoon diagrams of moCD163 SRCR5 represented in the 180° rotation. Seven β-strands β1-7, three helices α1-3 and the loop regions are colored in blue, magenta and cyan, respectively. The N- and C-termini are labeled. **B** The ribbon diagrams of moCD163 SRCR5 showing the disulfide bonds represented in the 180° rotation. The disulfide bonds are colored in yellow and the bridged cysteines are labeled. **C** Structural comparison of moCD163 SRCR5 with pCD163 SRCR5. The crystal structures of moCD163 SRCR5 was aligned with pCD163 SRCR5 in cartoon diagrams. The pCD163 SRCR5 and moCD163 SRCR5 are in green and cyan, respectively. Their N- and C-termini are labeled, and their minor differences are circled in dashed lines.

**Figure 2:**
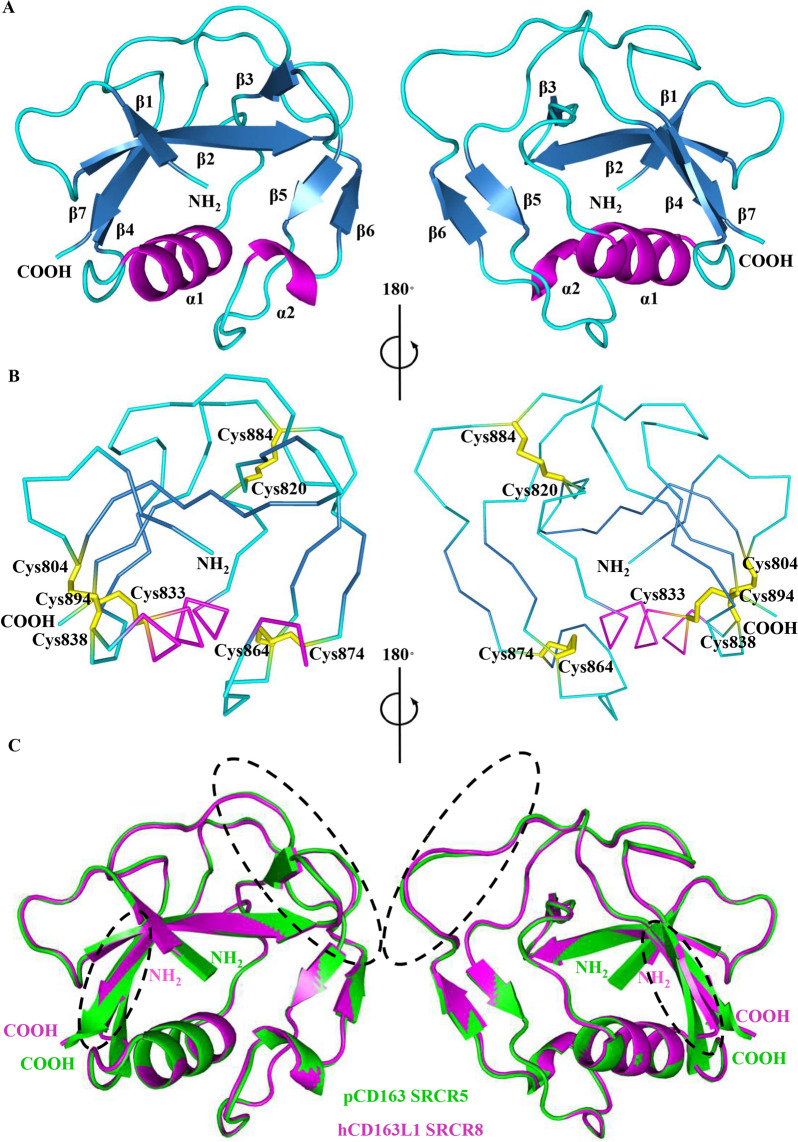
**Crystal structure of hCD163L1 SRCR8 and comparison with pCD163 SRCR5. A** The cartoon diagrams of hCD163L1 SRCR8 represented in the 180° rotation. Seven β-strands β1-7, two helices α1-2 and the loop regions are colored as in Figure 1A. The N- and C-termini are labeled. **B** The ribbon diagrams of hCD163L1 SRCR8 showing the disulfide bonds represented in the 180° rotation as in Figure 1B. **C** Structural comparison of hCD163L1 SRCR8 with pCD163 SRCR5.The crystal structures of hCD163L1 SRCR8 was aligned with pCD163 SRCR5 in cartoon diagrams. The pCD163 SRCR5 and hCD163L1 SRCR are in green and magenta, respectively. Their N- and C-termini are labeled, and their differences are circled in dashed lines.

In Figures 1A and 2A, both moCD163 SRCR5 and hCD163L1 SRCR8 adopted a compact heart shape and were characterized by the presence of a long flexible loop region. MoCD163 SRCR5 was comprised of two antiparallel β-sheets (β1-4, β7 and β5-6) and three helices (a single α-helix α2 and two 3_10_-helices α1, α3), whereas hCD163L1 SRCR8 contained two antiparallel β-sheets (β1-4, β7 and β5-6) and two helices (a single α-helix α1 and a 3_10_-helix α2). Four disulfide bonds were bridged in moCD163 SRCR5 (Cys487-Cys521, Cys503-Cys567, Cys516-Cys577 and Cys547-Cys557, the numbering is according to UniProt entry Q2VLG4, Figure 1B) and hCD163L1 SRCR8 (Cys804-Cys838, Cys820-Cys884, Cys833-Cys894 and Cys864-Cys874, the numbering is according to UniProt entry Q9NR16, Figure 2B), consistent with the typical disulfide linkage pattern of class B SRCR [34].

### Structural comparison of moCD163 SRCR5 or hCD163L1 SRCR8 with pCD163 SRCR5

As shown in Figures 1C and 2C, a close comparison of moCD163 SRCR5 or hCD163L1 SRCR8 with pCD163 SRCR5 revealed that they shared almost identical structural folds (RMSD = 0.355 Å for 79 matching Cα atoms between moCD163 SRCR5 and pCD163 SRCR5, RMSD = 0.249 Å for 95 matching Cα atoms between hCD163L1 SRCR8 and pCD163 SRCR5). The minor differences between moCD163 SRCR5 and pCD163 SRCR5 were only observed in the long loop regions (Figure 1C). In addition to minor differences in the long loop region, hCD163L1 SRCR8 contained a shorter β4 (Ile842-Ser845) than that of pCD163 SRCR5 (Thr522-Leu527) (Figure 2C).

**Figure 3:**
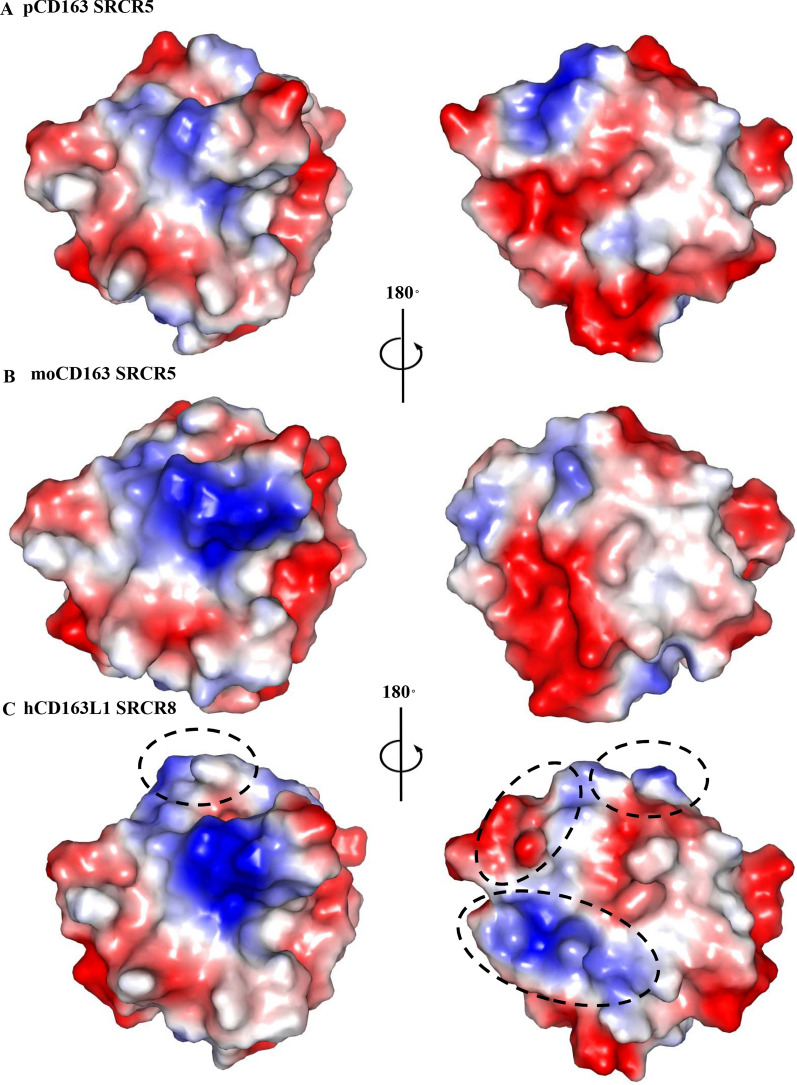
**Surface electrostatic potentials of pCD163 SRCR5 (A), moCD163 SRCR5 (B) and hCD163L1 SRCR8 (C).** The figures are represented in the 180° rotation to depict basic residues in the positively-charged clusters (blue) and acidic residues in the negatively-charged areas (red). The differences of hCD163L1 SRCR8 from the other two proteins are indicated with dashed circles. The electrostatic potentials are colored from − 62 to + 62 kiloteslas/charge.

**Figure 4:**
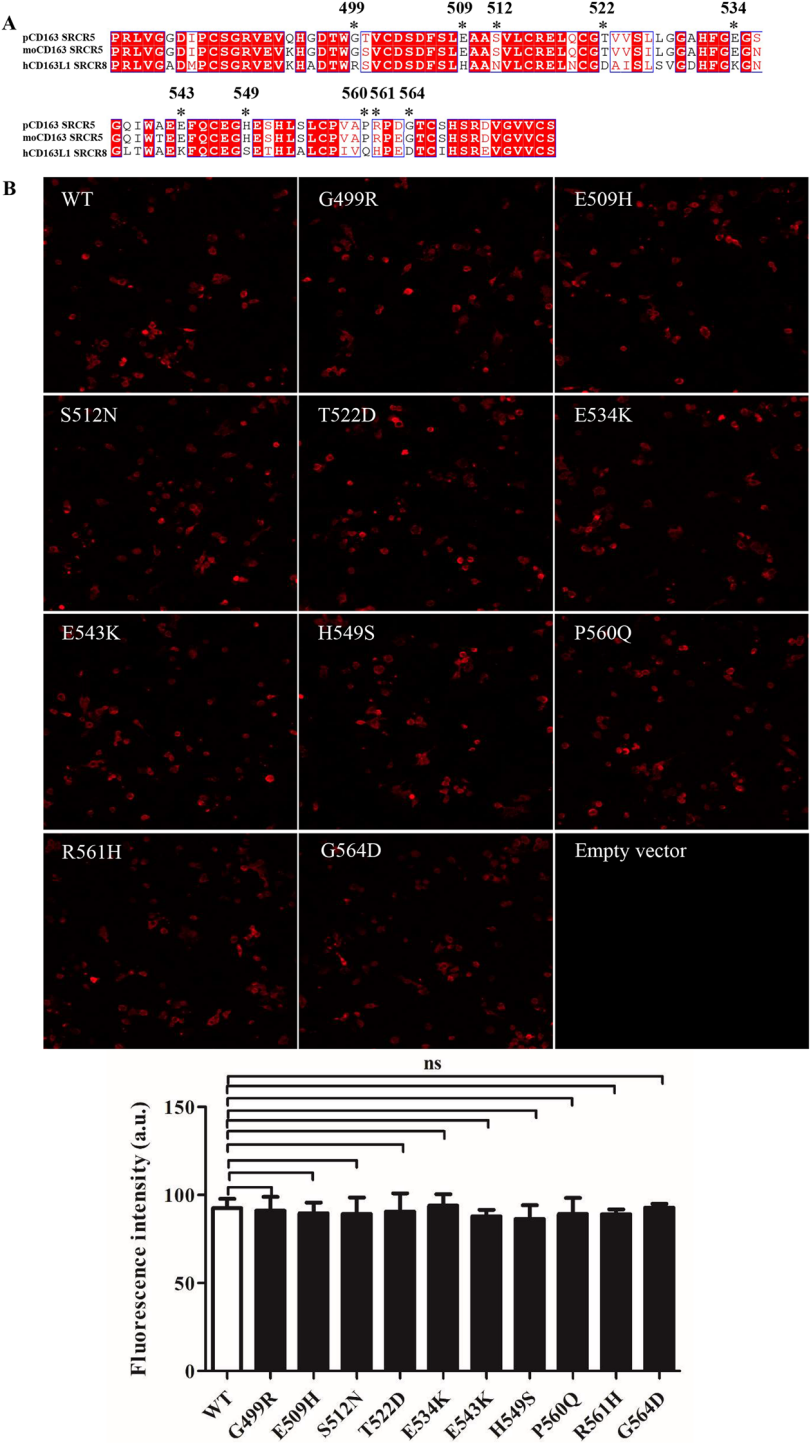
**IFA analyses of WT and mutant pCD163 expression. A** Sequence alignment of pCD163 SRCR5, moCD163 SRCR5 and hCD163L1 SRCR8. The amino acid sequences of pCD163 SRCR5 (UniProt entry Q2VL90), moCD163 SRCR5 (UniProt entry Q2VLG4) and hCD163L1 SRCR8 (UniProt entry Q9NR16) are aligned. The chosen mutated residues are marked by asterisks (*). **B** IFA analyses of WT and mutant pCD163 expression. The PK-15 cells were transfected with WT or pCD163 mutated constructs (1 μg/well, 24-well plate). Twenty four hours post-transfection, the cells were fixed and stained with a commercial mouse anti-pCD163 monoclonal antibody (MCA2311GA), and then examined by IFA. The total fluorescence intensity of pCD163 was calculated using ImageJ software. Data represent means ± SEM of three independent experiments. ns, not significant.

As shown in Figures 3A and B, moCD163 SRCR5 displayed a similar surface electrostatic potential as pCD163 SRCR5 did, so-called “D/E-R-rich” charge distribution [40]. In contrast, hCD163L1 SRCR8 showed a significantly different surface electrostatic potential from the other two proteins (Figure 3). There were more positively and negatively charged regions in hCD163L1 SRCR8 due to different amino acid contents (Figures 3 and 4A). Especially, certain regions with the opposed charge were clearly observed (Figure 3), where the acidic residues were replaced by the basic ones (Figure 4A). Based on the comparison, we hypothesize that the difference in the surface electrostatic potentials between hCD163L1 SRCR8 and pCD163 SRCR5 may result in reduced cell permissiveness to PRRSV-2 infection, rather than their structural folds.

### Site-directed mutagenesis of SRCR5 in pCD163

**Figure 5:**
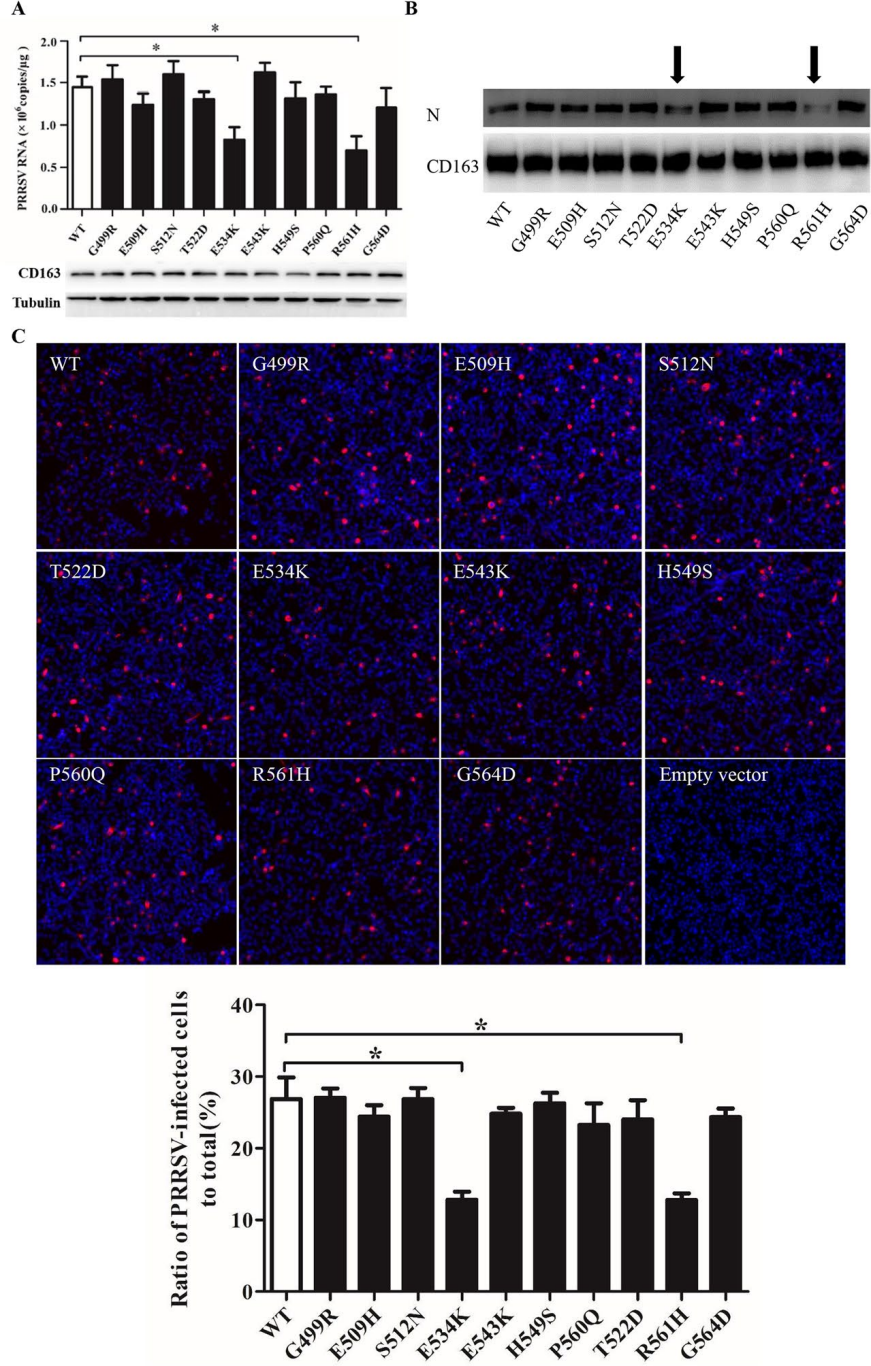
**Identification of residues in pCD163 SRCR5 important for PRRSV-2 infection. A** RT-qPCR analyses of the effect of mutated pCD163 on PRRSV-2 replication. WT or mutant pCD163 constructs (3 μg/well, 6-well plate) were transfected into PK-15 cells. After 24 h, the transfected PK-15 cells were inoculated with PRRSV-2 strain BJ-4 at a MOI of 1. At 12 hpi, total RNAs of infected PK-15 cells were extracted and then the viral RNA was measured by RT-qPCR. In parallel, expression levels of WT and mutant pCD163 were tested by IB using a commercial mouse anti-human CD163 antibody (MCA1853). Data represent means ± SEM from three independent experiments. *p < 0.05. **B** Analyses of PRRSV N protein expression in WT or mutant pCD163-expressed cells. The infected WT or mutant pCD163-expressed cells were harvested and lysed at 24 hpi. PRRSV N protein in WT or mutant pCD163-expressed cells was measured by IB. Significant reduction in PRRSV N protein expression is marked by an arrow. In parallel, expression levels of WT and mutant pCD163 were tested by IB using a commercial mouse anti-human CD163 antibody (MCA1853). **C** PRRSV infectivity in WT or mutant pCD163-expressed cells. The infected cells (24 hpi) were fixed and stained with PRRSV N protein (red) antibody. Nuclei were stained with DAPI and examined by IFA. The total fluorescence intensity of PRRSV N protein was calculated using ImageJ software. Data represent means ± SEM of three independent experiments. *p < 0.05.

Based on the analyses above, we chose the residues Gly499, Glu509, Ser512, Thr522, Glu534, Glu543, His549, Pro560, Arg561 and Gly564 in pCD163 SRCR5, whose surface electrostatic potential was significantly different from that in hCD163L1 SRCR8. We mutated each residue to the corresponding one in hCD163L1 SRCR8, namely G499R, E509H, S512N, T522D, E534K, E543K, H549S, P560Q, R561H and G564D (Figure 4A). WT or each mutated CD163 construct was transfected into PK-15 cells. Native PK-15 cells express no pCD163 and are refractory to PRRSV infection, while they are permissive to the viral infection after transfection with pCD163 [40]. After 24 h, the transfected PK-15 cells were monitored by IFA with a commercial anti-CD163 antibody. As shown in Figure 4B, all mutated and WT pCD163 receptors were expressed at almost the same level at 24 h post-transfection in PK-15 cells. In addition, IB detected their identical expression patterns at 36 h and 48 h post-transfection in PK-15 cells in Figures 5A and B, respectively. These results ruled out the influence of their expression levels in our subsequent experiments.

### Identification of residues in pCD163 SRCR5 important for PRRSV-2 infection

**Figure 6:**
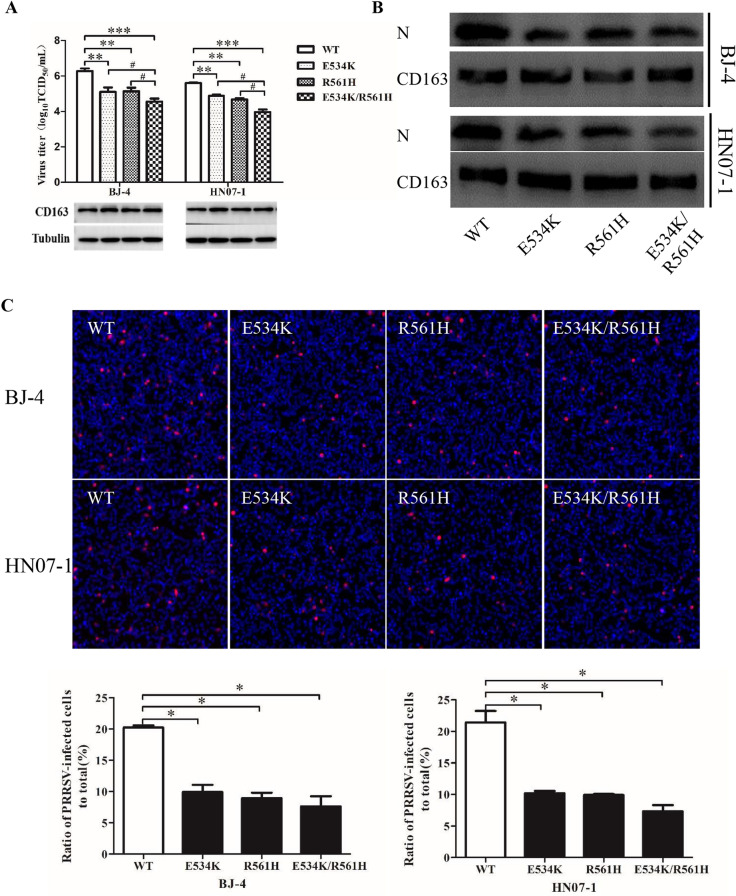
**Analyses of simultaneous mutagenesis of the identified two residues for PRRSV-2 infection. A** TCID_50_ analyses of the effect of mutated CD163 on PRRSV-2 viral titers. PK-15 cells were transfected with WT or mutated pCD163 constructs. After 24 h, the transfected PK-15 cells were inoculated with PRRSV strain BJ-4 or HN07-1 at a MOI of 1 for 48 h. The viral yields were measured by TCID_50_ assay in MARC-145 cells. Data represent means ± SEM of three independent experiments. **p < 0.01, ***p < 0.001 for mutant CD163 compared to the WT one. ^#^*p* < 0.05 for simultaneous mutagenesis at position 534 and 561 of CD163 compared to the single-site mutagenesis. **B** Analyses of PRRSV N protein expression in WT or mutant pCD163-expressed cells. PRRSV N protein expression was tested by IB as described in Figure 5B. **C** PRRSV infectivity in WT or mutant pCD163-expressed cells. The infectivity was measured by IFA as described in Figure 5C. Data represent means ± SEM of three independent experiments. *p < 0.05.

In order to detect the effect of each mutated residue on PRRSV-2 infection, we first utilized a typical PRRSV-2 VR2332-like strain BJ-4 to measure viral infections. As shown in Figure 5A, RT-qPCR analysis demonstrated that the transfected cells with the mutated construct G499R, E509H, S512N, T522D, E543K, H549S, P560Q or G564D showed comparable PRRSV RNA abundance as the WT construct-transfected ones did (*p* > 0.05). In contrast, the transfected cells showed 0.72 × 10^6^ copies/μg in viral RNA abundance with site-directed mutagenesis of pCD163 at position 534 and 0.65 × 10^6^ copies/μg at position 561, respectively, corresponding to > 50% reduction compared to the WT ones (1.45 × 10^6^ copies/μg). These results of RT-qPCR showed statistically significant in reduction of viral RNA abundance (*p* < 0.05). In addition, in Figures 5B and C, compared to the WT one, the mutated pCD163 at position 534 or 561 showed a strong inhibitory effect on PRRSV N protein expression and infectivity (> 50%; *p* < 0.05). The TCID_50_ results further corroborated the importance of the residue 534 or 561 for PRRSV-2 infection, where the progeny virus titers were decreased by more than tenfold (~5.1 log_10_TCID_50_/mL for the mutated pCD163 at position 534 or 561, compared to 6.3 log_10_TCID_50_/mL for the WT one; namely > 1 log_10_TCID_50_/mL, *p* < 0.01; Figure 6A).

### Analyses of simultaneous mutagenesis of the identified two residues for PRRSV-2 infection

Next, we examined the effect of simultaneous mutagenesis of the identified two residues on PRRSV-2 infection. As shown in Figure 6A, TCID_50_ analysis demonstrated that the transfected cells with simultaneous mutagenesis at position 534 and 561 showed an additive decrease in the viral titer compared with the single-site mutagenesis of pCD163 at position 534 or 561 (*p* < 0.05). Importantly, compared to the WT one (6.3 log_10_TCID_50_/mL), simultaneous mutagenesis (4.4 log_10_TCID_50_/mL) showed an almost 100-fold decreased viral titer (~2 log_10_TCID_50_/mL; *p* < 0.001). We also utilized a HP-PRRSV strain, HN07-1, to carry out the viral titration assay and observed similar results (Figure 6A). Furthermore, we measured PRRSV N protein expression and infectivity to test the effect of simultaneous mutagenesis on viral infection. As shown in Figures 6B and C, IB and IFA analyses demonstrated that simultaneous mutagenesis showed a significant reduction in PRRSV infection compared with the WT one (*p* < 0.05).

**Figure 7:**
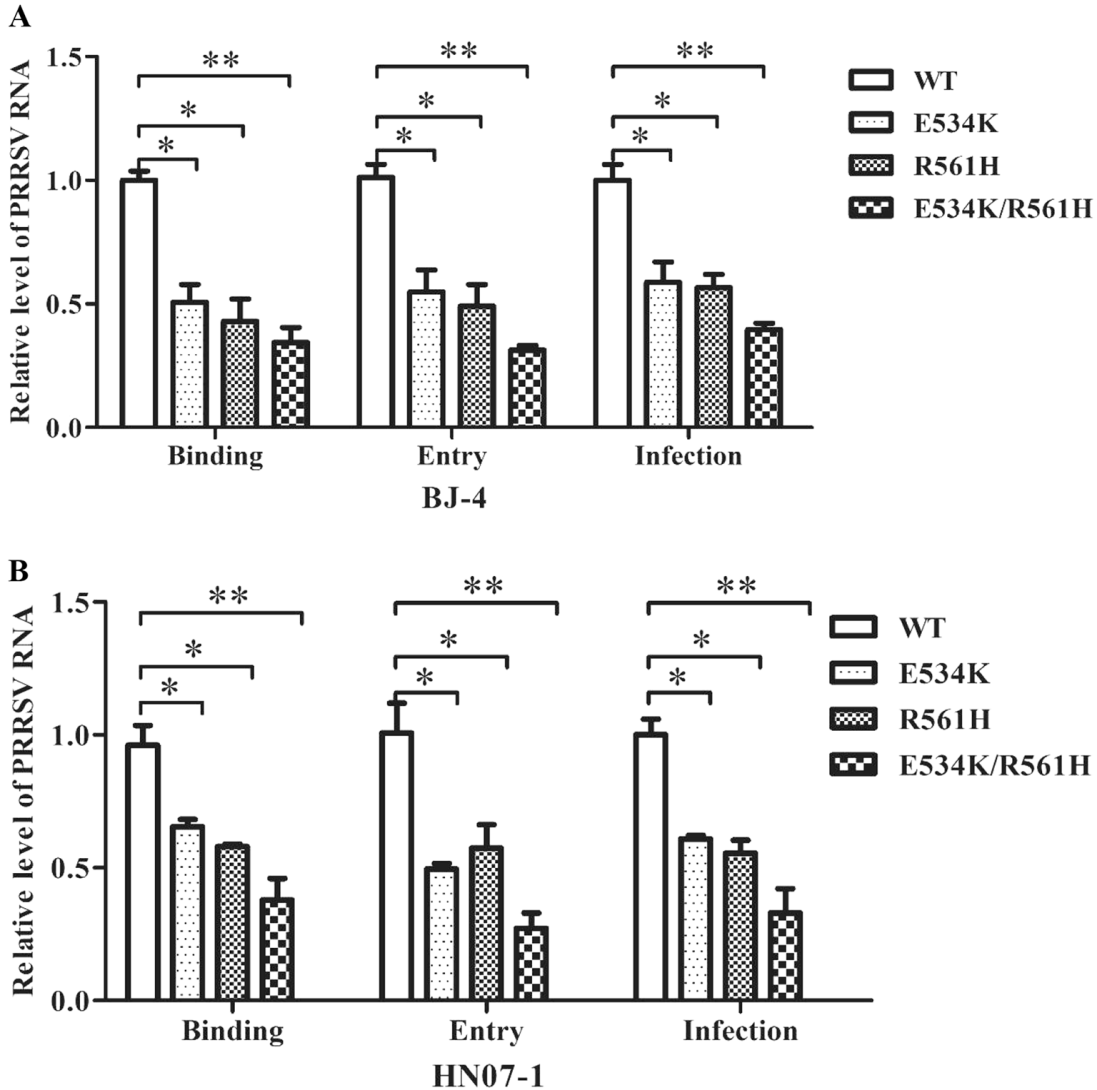
**Analyses of the effect of the mutated pCD163 on PRRSV**-**2 invasion. A**, **B** The binding, entry and infection assays with mutated pCD163 for PRRSV-2 strain BJ-4 and HN07-1, respectively. WT or mutated pCD163 constructs were transfected into PK-15 cells. After 24 h, the transfected PK-15 cells were inoculated with PRRSV strain BJ-4 or HN07-1 at a MOI of 1 at 4 °C for 1 h, and then relative quantitation of viral RNA abundance was carried out. For PRRSV entry assay, the unbound viruses were washed away and the inoculated cells were then cultured at 37 °C for 3 h to allow viral entry. The entering viral RNA was analyzed by RT-qPCR. For PRRSV infection, PRRSV strain BJ-4 or HN07-1 was inoculated in the transfected PK-15 cells as described above. PRRSV RNA abundance was tested by RT-qPCR. For the RT-qPCR, PRRSV ORF7 gene was normalized with GAPDH mRNA and relatively quantified by the 2^−ΔΔCT^ method. In parallel, we have transfected empty vector and inoculated PRRSV as negative control. However, the C(t) value of the negative control was comparable to that of water, which was not included in the manuscript. Data represent means ± SEM of three independent experiments. *p < 0.05, **p < 0.01.

Finally, we explored how these residues influenced PRRSV-2 infection through viral binding and entry assays. The results showed that these two residues actually took effect during the viral binding stage (Figure 7).

All these results demonstrated that residues 534 and 561 were important for PRRSV-2 infection in vitro.

## Discussion

Heparin sulfate [61, 62], sialoadhesin (Sn/CD169) [63, 64], CD163 [25], CD151 [65], vimentin [66], dendritic cell-specific intercellular adhesion molecule-3-grabbing nonintegrin [67] and non-muscle myosin heavy chain 9 [60] were previously proposed to be associated with PRRSV infection. Among them, Sn and CD163 have been intensively studied; the former was reported to be responsible for viral attachment and internalization, and the latter contributed to viral membrane fusion and uncoating [27]. However, Sn knockout pigs were still susceptible to PRRSV infection [68]. Increasing evidence supports the view that CD163 is an indispensable receptor and its SRCR5 domain is crucial for PRRSV infection both in vitro and in vivo [25, 30–33, 36, 37, 39, 69–71]. However, the detailed mechanisms of CD163, especially the SRCR5, involved in PRRSV infection are not fully understood.

In this study, we determined the crystal structures of moCD163 SRCR5 and hCD163L1 SRCR8 (Figures 1 and 2). Compared to the crystal structure of pCD163 SRCR5, these three SRCR domains shared almost identical structural folds (Figures 1C and 2C). Intriguingly, hCD163L1 SRCR8 showed a significantly different surface electrostatic potential compared to the other two proteins (Figure 3). The comparison provided a structural basis to explain why stable expression of moCD163 rendered comparable infectivity to PRRSV as pCD163 did, since their crucial SRCR5 shared the overall conformations and surface electrostatic potentials [25]. Furthermore, this comparison suggested that the surface electrostatic potential might lead to different cell susceptibility to PRRSV infection between pCD163 and hCD163L1 [31, 39], where pCD163 SRCR5 substitution by homologous hCD163L1 SRCR8 showed a significantly reduced permissiveness to PRRSV-2. Therefore, we focused on the surface electrostatic potential differences between pCD163 SRCR5 and hCD163L1 SRCR8.

We carried out mutational studies to identify that the residue at position 561 was important for PRRSV-2 infection in vitro (Figures 5 and 6). The arginine residue at position 561 (Arg561) was reported to participate during viral infection in our previous study [40]. In addition, we identified a novel residue at position 534 important for PRRSV-2 infection (Figures 5 and 6). Moreover, both these two residues influenced the viral invasion process (Figure 7). Importantly, simultaneous mutagenesis of these two residues conferred additive resistance to PRRSV-2 infection in vitro as shown in Figure 6. These results demonstrate that the two residues showed a biological significance regarding PRRSV-2 actual infection and the charge may contribute to their involvement. Of course, whether the in vitro results will be applicable to the in vivo viral infection needs further demonstration. For example, although PAMs from the genetically modified pigs with hCD163L1 SRCR8 substitution showed a significantly reduced permissiveness, the corresponding pigs were resistant to PRRSV-1 and HP-PRRSV, but not to typical PRRSV-2 [31, 39]. The underlying mechanisms are interesting to be explored. Additionally, whether these two residues are important for PRRSV-1 infection should be addressed. It is worth mentioning that in addition to SRCR5, other SRCR domains of CD163 may be involved in PRRSV infection, which will be addressed in the future.

In fact, we have tried to determine the crystal structure of human CD163 (hCD163) SRCR5. Unexpectedly, recombinant hCD163 SRCR5, moCD163 SRCR5 and hCD163L1 SRCR8 showed a different expression pattern in the same expression system despite their high sequence identities (data not shown). Furthermore, crystals of hCD163 SRCR5 diffracted to a low quality and was unable to be processed although it was successfully prepared in *Drosophila* S2 cells and crystallized under the same condition as those of hCD163L1 SRCR8 (data not shown). All these phenomena are attractive to clarify.

In conclusion, we have compared the crystal structures among pCD163 SRCR5, moCD163 SRCR5 and hCD163L1 SRCR8. Based on the structural comparison, we identified that the charged residue at position 534 in association with the one at position 561 in the long loop region were important for PRRSV-2 infection in vitro. The results provide clues for CD163-mediated PRRSV infection and deepen our understanding of the viral pathogenesis, which will support the genome-edited implications to select pigs resistant to PRRSV.

## Data Availability

The coordinates of moCD163 SRCR5 and hCD163L1 SRCR8 were deposited in the Protein Data Bank (PDB code 6K0L and 6K0O).

## Abbreviations

PRRSV: Porcine reproductive and respiratory syndrome virus
SRCR: Scavenger receptor cysteine-rich domain
HP-PRRSVs: Highly pathogenic variants of PRRSV-2
moCD163: Monkey CD163
hCD163L1: Human CD163-like homolog
pCD163: Porcine CD163
PAMs: Porcine alveolar macrophages
NLFK: Norden Laboratories feline kidney
BHK-21: Baby hamster kidney-21
TCID_50_: 50% Tissue culture infective dose
hCD163: Human CD163
RIPA: Radio immunoprecipitation assay
PDB: Protein Data Bank
SEM: Standard errors of the means
GAPDH: Glyceraldehyde-3-phosphate dehydrogenase
PFA: Paraformaldehyde
PK: Porcine kidney
SSRF: Shanghai Synchrotron Radiation Facility
